# Machine learning-based comprehensive analysis of m6A RNA methylation regulators in colorectal cancer: implications for prognosis, immune microenvironment, and immunotherapy response

**DOI:** 10.3389/ebm.2025.10776

**Published:** 2026-01-14

**Authors:** Feifei Kong, Jiawei Feng, Haixia Shan, Youlong Zhu, Ling-Jun Zhu

**Affiliations:** 1 Department of Oncology, The First Affiliated Hospital of Nanjing Medical University, Nanjing, China; 2 Department of Oncology, The Affiliated Hospital of Xuzhou Medical University, Xuzhou, Jiangsu, China; 3 Department of Thyroid Surgery, The Third Affiliated Hospital of Soochow University, Changzhou First People’s Hospital, Changzhou, Jiangsu, China; 4 Department of Gastrointestinal Surgery, Southeast University Affiliated Xuzhou Central Hospital, Xuzhou, Jiangsu, China

**Keywords:** colorectal cancer, immunotherapy, m6A methylation, machine learning, SHAP

## Abstract

N6-methyladenosine (m6A) RNA methylation regulators have been implicated in colorectal cancer (CRC) progression. However, systematic evaluation using multiple machine learning approaches for prognostic prediction remains limited. This study aimed to develop and validate machine learning models for CRC prognosis based on m6A regulators and assess their potential for immunotherapy response prediction. We analyzed 1,047 CRC patients from TCGA and GEO databases (70% training, 30% validation). Twenty machine learning algorithms were systematically evaluated, with LASSO regression selecting optimal features from 27 m6A regulators. SHAP analysis provided model interpretability. Immune microenvironment characterization and immunotherapy response prediction were performed using established computational methods. LASSO regression selected eight m6A regulators (IGF2BP2, METTL3, HNRNPA2B1, METTL14, YTHDF2, VIRMA, FTO, ALKBH5) for model construction. Among 20 algorithms tested, Random Forest achieved optimal performance (training AUC = 0.895, validation AUC = 0.847). SHAP analysis identified IGF2BP2 (mean |SHAP| = 0.42) and METTL3 (mean |SHAP| = 0.36) as primary contributors to risk prediction. Risk stratification showed significant survival differences (HR = 2.41, 95% CI: 1.73–3.36, p < 0.001). Low-risk patients demonstrated enhanced immune infiltration with higher CD8^+^ T cells (17.8% vs. 10.2%, p < 0.001) and better predicted immunotherapy response rates (36.5% vs. 20.3%, p = 0.006). Our systematic machine learning analysis demonstrates that m6A regulators can effectively predict CRC prognosis and immunotherapy response. The eight-gene signature provides a practical tool for clinical risk assessment and treatment decision-making.

## Impact statement

This study addresses the need for reliable prognostic tools in colorectal cancer by systematically evaluating machine learning approaches for m6A-based risk stratification. While m6A modifications are increasingly recognized in cancer biology, their clinical application remains limited by methodological inconsistencies. We advance the field by providing the first comprehensive comparison of 20 ML algorithms for m6A-based CRC prognosis, establishing a standardized framework for future studies. Our integration of SHAP analysis addresses the critical barrier of model interpretability in clinical settings. The resulting 8-gene signature demonstrates potential utility for patient stratification and preliminary evidence for immunotherapy response prediction. This work provides the research community with a validated methodology for developing m6A-based biomarkers and offers clinicians a potential tool for risk assessment. The findings contribute to the growing understanding of m6A’s role in CRC progression and immune regulation, supporting further investigation into epigenetic-based therapeutic strategies.

## Introduction

Colorectal cancer (CRC) remains the third most common malignancy worldwide, with over 1.9 million new cases and more than 900,000 deaths annually reported in 2024 [1]. Despite significant advances in surgical techniques, chemotherapy, and targeted therapies, the 5-year survival rate for metastatic CRC remains approximately 14%, highlighting the urgent need for improved prognostic tools and personalized treatment strategies [2]. CRC’s diverse molecular subtypes and treatment responses require sophisticated predictive models to capture this complexity.

N6-methyladenosine (m6A) represents the most abundant internal chemical modification of eukaryotic mRNAs, accounting for approximately 0.1–0.4% of all adenosines in cellular mRNA [3]. This reversible modification regulates various aspects of RNA metabolism, including stability, translation efficiency, nuclear export, and localization [4]. The m6A modification is dynamically regulated by three categories of proteins: “writers” (methyltransferases such as METTL3, METTL14, WTAP), “readers” (binding proteins including YTHDF1/2/3, IGF2BP1/2/3, HNRNPC), and “erasers” (demethylases including FTO and ALKBH5) [5].

Accumulating evidence demonstrates that m6A dysregulation is causally involved in cancer initiation, progression, metastasis, and therapeutic resistance [6, 7]. In CRC specifically, recent mechanistic studies have elucidated the pathogenic roles of m6A regulators. Wang et al. demonstrated that HES1 promotes aerobic glycolysis through IGF2BP2-mediated GLUT1 m6A modification, driving CRC progression via m6A-dependent metabolic reprogramming [8]. Zhou et al. revealed that METTL3-mediated m6A modification promotes metastasis through REG1α stabilization and Wnt/β-catenin pathway activation, establishing a direct link between epigenetic modification and tumor progression [9]. Most recently, Qiao et al. showed that FTO demethylase targeting induces ferroptotic cell death through SLC7A11/GPX4 downregulation, highlighting therapeutic vulnerabilities [10]. METTL3 overexpression has been shown to promote CRC cell proliferation and metastasis through multiple mechanisms, including JAK1/STAT3 signaling activation and STC2 axis regulation [11, 12]. Conversely, enhanced m6A modification through demethylase inhibition has been associated with increased chemosensitivity and ferroptosis induction in CRC cells [13]. However, the comprehensive prognostic value of m6A regulators and their relationship with the tumor immune microenvironment in CRC remains incompletely understood.

Machine learning has transformed biomedical research and precision oncology by analyzing complex datasets to identify patterns and make predictions [14]. Unlike traditional statistical methods, machine learning algorithms can capture non-linear relationships and complex interactions between variables, making them suitable for analyzing the intricate regulatory networks of m6A modifications. However, the “black box” nature of complex machine learning models poses a significant barrier to clinical adoption, as physicians require transparent, interpretable predictions to make informed treatment decisions [15]. SHapley Additive exPlanations (SHAP), a unified framework based on cooperative game theory, has emerged as widely adopted method for model interpretation by quantifying each feature’s contribution to individual predictions [16]. SHAP analysis has demonstrated robust performance across diverse domains including transportation systems, autonomous vehicle security [17], maritime risk assessment [18], and critically, biomedical applications. In healthcare, SHAP has been successfully applied to predict sepsis outcomes [19], interpret deep learning models in radiology, and identify key genetic drivers in cancer prognosis. The method’s model-agnostic nature and consistency with human intuition make it particularly valuable for translating complex computational models into clinically actionable insights.

Previous studies have primarily focused on individual m6A regulators or utilized limited machine learning approaches for CRC prognosis prediction. To our knowledge, no study has comprehensively evaluated 20 different machine learning algorithms for m6A-based prognostic modeling in CRC, nor has any study systematically investigated the relationship between m6A-based risk stratification and immunotherapy response prediction.

**FIGURE 1:**
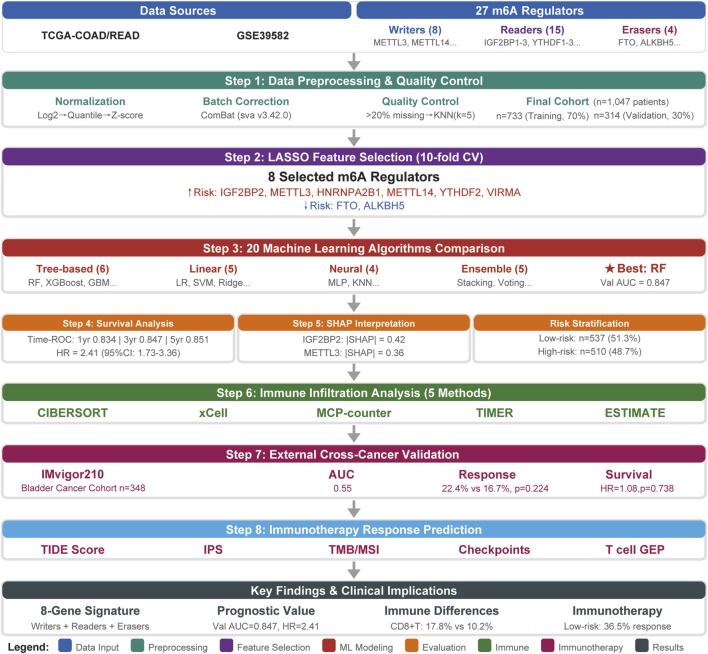
Comprehensive study workflow and analytical framework. The flowchart illustrates the complete analytical pipeline from data acquisition through clinical translation. (1) multi-cohort data acquisition (TCGA, GEO, validation cohorts); (2) data preprocessing and quality control; (3) feature selection via LASSO (27→8 m6A regulators); (4) machine learning model development with 20 algorithms; (5) model interpretation using SHAP analysis; (6) comprehensive validation across survival, immune microenvironment, and therapeutic response dimensions; (7) external validation in independent immunotherapy cohorts; and (8) development of clinical translation tools.

In this study, we aimed to: (1) develop and validate a comprehensive computational framework incorporating 20 machine learning algorithms for m6A-based CRC prognosis prediction using m6A regulators; (2) identify key m6A genes contributing to prognosis using LASSO feature selection; (3) provide model interpretability through SHAP analysis; (4) investigate the relationship between m6A-based risk stratification and immune microenvironment characteristics; and (5) evaluate the predictive value for immunotherapy response using established computational biomarkers. This work provides a hypothesis-generating framework to guide future experimental validation and clinical trials. Our findings provide a robust framework for personalized risk assessment and treatment selection in CRC patients. The overall study design and analytical workflow are illustrated in Figure 1.

## Materials and methods

### Study design and data sources

This retrospective study followed the Transparent Reporting of a multivariable prediction model for Individual Prognosis Or Diagnosis (TRIPOD) guidelines. We utilized publicly available gene expression and clinical data from The Cancer Genome Atlas (TCGA) COAD/READ cohorts and Gene Expression Omnibus (GEO) datasets (GSE39582).

### Data accessibility

TCGA COAD:^1^
TCGA READ:^2^
GEO GSE39582:^3^
IMvigor210:^4^

The combined dataset comprised 1,047 CRC patients with complete gene expression profiles and clinical follow-up data, representing one of the largest cohorts utilized for m6A-based prognostic modeling in CRC. This sample size substantially exceeds the minimum requirements for stable machine learning model development and provides adequate statistical power for our analyses.

**FIGURE 2:**
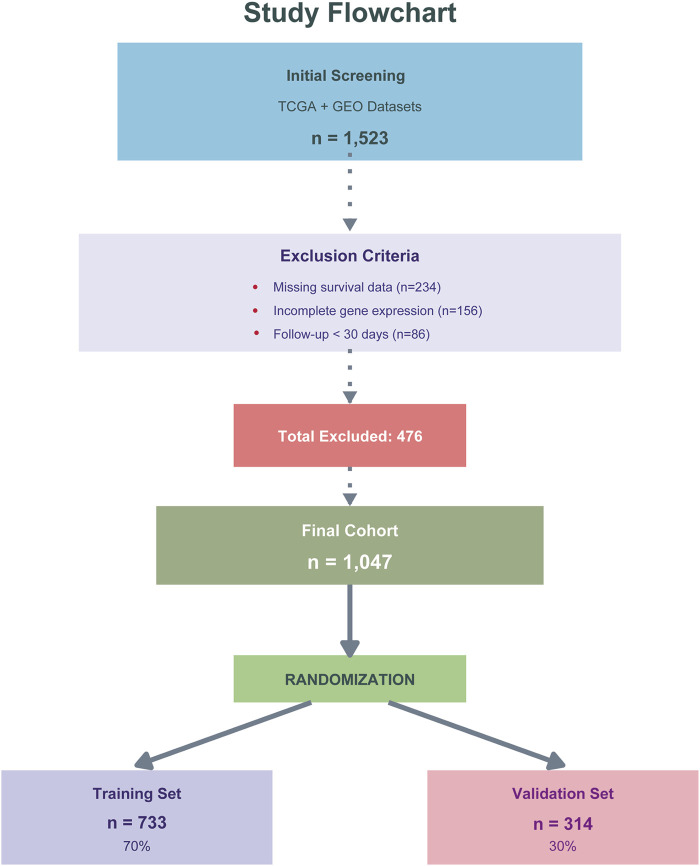
Study design and patient flow diagram. Flowchart showing patient selection from TCGA COAD/READ and GEO (GSE39582) databases. Of 1,523 initially screened patients, 476 were excluded due to missing survival data (n = 234), incomplete gene expression (n = 156), or follow-up <30 days (n = 86). The final cohort (n = 1,047) was randomly divided into training (n = 733, 70%) and validation (n = 314, 30%) sets.

The dataset was divided into training (n = 733, 70%) and validation (n = 314, 30%) cohorts using stratified random sampling to maintain balanced outcome distribution. Data from TCGA COAD/READ and GEO dataset GSE39582 were first combined and then randomly split, with the training cohort used for model development, feature selection, and hyperparameter optimization via 5-fold cross-validation, while the validation cohort served as an independent holdout set for unbiased performance evaluation. The inclusion/exclusion criteria and screening process are detailed in Figure 2. All data were obtained from public repositories with appropriate ethical approvals from the original studies, and this secondary analysis was exempt from additional institutional review board approval.

### m6A regulators and data preprocessing

We identified 27 m6A regulators through systematic literature review and functional annotation databases, comprising 8 writers (METTL3, METTL14, WTAP, VIRMA, RBM15, RBM15B, ZC3H13, ZCCHC4), 4 erasers (FTO, ALKBH5, CBLL1, ELAVL1), and 15 readers (YTHDC1, YTHDC2, YTHDF1-3, HNRNPC, FMR1, LRPPRC, HNRNPA2B1, IGFBP1-3, IGF2BP1-3).

Gene expression data underwent sequential preprocessing: log2 transformation, quantile normalization using the preprocessCore R package, and Z-score standardization within each dataset. Multi-source data integration employed Combat batch correction (sva R package version 3.42.0). Quality control removed genes with >20% missing values, followed by k-nearest neighbors imputation (k = 5) using the VIM R package. Clinical variables included age at diagnosis, gender, tumor stage (AJCC 8th edition), tumor location, microsatellite instability status, and survival outcomes (overall survival time and vital status).

### Feature selection and model development

LASSO regression with 10-fold cross-validation identified prognostically relevant m6A regulators using the glmnet R package (version 4.1-4). The optimal lambda parameter was selected using the one standard error rule (lambda.1se) with random seed set to 123 for reproducibility. Twenty machine learning algorithms were implemented in Python 3.8 using scikit-learn (version 1.0.2), XGBoost (version 1.6.1), LightGBM (version 3.3.2), and CatBoost (version 1.0.6). Hyperparameter optimization employed 5-fold stratified cross-validation with grid search (random_state = 42). Class imbalance was addressed using SMOTE from the imbalanced-learn package (version 0.8.1) with random_state = 42.

### Model evaluation and interpretability

Model performance was assessed using AUC-ROC as the primary metric, complemented by AUC-PR, accuracy, sensitivity, specificity, precision, F1-score, and Matthews correlation coefficient calculated using scikit-learn metrics. Model calibration was evaluated using Hosmer-Lemeshow test (scipy.stats) and calibration plots.

We selected SHAP (SHapley Additive exPlanations) as our primary interpretability framework based on several key advantages. First, SHAP is grounded in cooperative game theory with solid mathematical foundations, uniquely satisfying three desirable properties: local accuracy, missingness, and consistency [15, 16]. Second, SHAP provides both individual-level explanations and global interpretability through aggregated SHAP values, which is critical for personalized medicine. Third, TreeExplainer enables computationally efficient calculation of exact SHAP values for tree-based models in polynomial time, making it feasible for clinical deployment. Finally, SHAP has been extensively validated in healthcare applications and demonstrates high physician acceptance due to its alignment with clinical reasoning patterns.

SHAP framework (version 0.40.0) provided model interpretability through TreeExplainer for tree-based models and KernelExplainer for others, generating feature importance rankings, waterfall plots, and interaction analyses for the optimal model.

### Risk stratification and survival analysis

Risk scores were calculated as weighted linear combinations of selected m6A regulators using LASSO coefficients: Risk Score = ∑(β_*i*_ × Gene_*i*_). Optimal cutoffs were determined via maximally selected rank statistics using the maxstat R package with minprop = 0.1 and maxprop = 0.9. Survival analysis employed Kaplan-Meier curves with log-rank tests (survival R package), Cox proportional hazards regression (coxph function), time-dependent ROC analysis using the timeROC R package for 1-, 3-, and 5-year predictions, concordance index (Harrell’s C-index), and restricted mean survival time (survRM2 R package).

### Immune microenvironment characterization

Tumor immune microenvironment was characterized using established algorithms: CIBERSORT (22 immune cell types) with LM22 signature matrix and 1,000 permutations, ESTIMATE algorithm for immune and stromal scores, MCP-counter for 10 immune and stromal populations, quanTIseq for immunotherapy-relevant cell types, and EPIC for immune and cancer cell fraction estimation. All analyses were performed using respective R packages with default parameters. Immune checkpoint genes (PDCD1, CD274, CTLA4, LAG3, HAVCR2, TIGIT) expression levels were extracted and log2-transformed. Immune phenotypes were classified as immune-inflamed (CD8^+^ T cells > median and immune score > median), immune-excluded (moderate immune infiltration), or immune-desert (both CD8^+^ T cells and immune score < median).

### Immunotherapy response prediction

Immunotherapy response potential was evaluated using established computational methods. Tumor mutational burden (TMB) was calculated as the total number of nonsynonymous mutations per megabase from somatic mutation data. Microsatellite instability (MSI) status was determined using MSIsensor algorithm with default parameters (≥3.5 classified as MSI-high). Neoantigen load was predicted using NetMHCpan 4.0 for HLA class I binding prediction with binding affinity threshold <500 nM. TIDE score was calculated using the TIDE web portal^5^. Immunophenoscore (IPS) was calculated based on four categories of genes (effector cells, immunosuppressive cells, MHC molecules, and checkpoints) using established methodology. T cell-inflamed gene expression profile (GEP) was calculated using the 18-gene signature with weighted sum approach.

### Cross-cancer validation

To evaluate the generalizability of our m6A risk model across different cancer types, we performed an independent cross-cancer validation using the IMvigor210 bladder cancer cohort. The IMvigor210 dataset comprises 348 patients with metastatic urothelial carcinoma who received atezolizumab (anti-PD-L1) immunotherapy, with available gene expression data, survival outcomes, and treatment response information. Gene expression data were log2-transformed and Z-score normalized. The eight m6A regulators from our CRC-derived model were mapped to the bladder cancer expression matrix. Risk scores were calculated using the fixed LASSO coefficients derived from the CRC training cohort, without any re-training. Patients were stratified into high-risk and low-risk groups based on the median risk score.

### Statistical analysis

All statistical analyses were performed using R (version 4.2.0) and Python (version 3.8). Continuous variables were compared using Student's t-test or Mann-Whitney U test based on normality assessed by Shapiro-Wilk test. Categorical variables were compared using chi-square test or Fisher’s exact test. Survival differences were assessed using log-rank test. Statistical significance was set at P < 0.05. Multiple testing correction was applied using Benjamini-Hochberg false discovery rate when appropriate. All computational analyses were performed with reproducible seeds to ensure result reproducibility.

Complete analysis code, detailed parameter settings, software environment specifications, and step-by-step workflow documentation are provided in the Supplementary Material (Supplementary Material S1, Supplementary Tables S1, S2). All analyses were performed with random seed = 42 to ensure reproducibility.

## Results

### Baseline characteristics

The study cohort comprised 1,047 CRC patients with a median age of 66 years [interquartile range (IQR): 57–74 years]. The training cohort (n = 733) included 392 males (53.5%) and 341 females (46.5%), while the validation cohort (n = 314) consisted of 171 males (54.5%) and 143 females (45.5%). Baseline characteristics were well-balanced between cohorts (Table 1).

**TABLE 1:** Baseline characteristics of study cohorts.

Characteristic	Training Set (n = 733)	Validation Set (n = 314)	P-value
Age, median (IQR)	66 (57–74)	67 (58–75)	0.542
Gender, n (%)			0.812
Male	392 (53.5%)	171 (54.5%)	
Female	341 (46.5%)	143 (45.5%)	
TNM Stage, n (%)			0.753
Stage I	127 (17.3%)	52 (16.6%)	
Stage II	276 (37.7%)	115 (36.6%)	
Stage III	243 (33.2%)	108 (34.4%)	
Stage IV	87 (11.9%)	39 (12.4%)	
Tumor Location, n (%)			0.834
Right colon	284 (38.7%)	125 (39.8%)	
Left colon	271 (37.0%)	112 (35.7%)	
Rectum	178 (24.3%)	77 (24.5%)	
MSI Status, n (%)			0.689
MSI-H	89 (12.1%)	41 (13.1%)	
MSS/MSI-L	644 (87.9%)	273 (86.9%)	
Adjuvant Chemotherapy, n (%)	423 (57.7%)	186 (59.2%)	0.674
Death Events, n (%)	200 (27.3%)	86 (27.4%)	0.973
Follow-up Time, median (IQR), months	32.5 (18.2–54.3)	31.8 (17.5–53.6)	0.721

### Feature selection of m6A regulators for prognostic model construction

**FIGURE 3:**
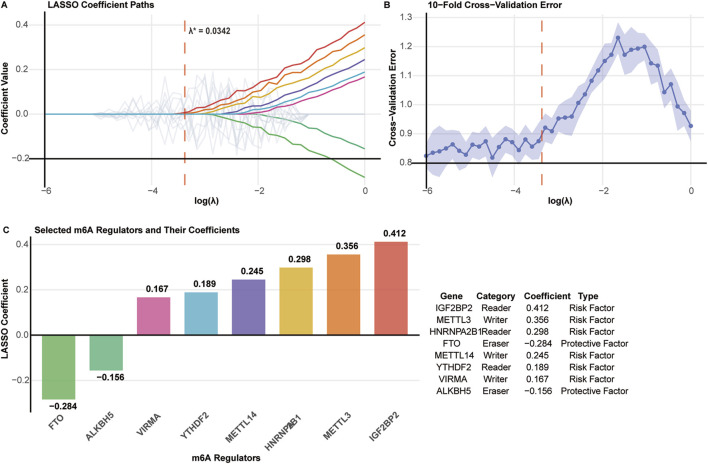
Feature selection of m6A regulators using LASSO regression. **(A)** LASSO coefficient paths for 27 m6A regulators. The optimal λ* = 0.0342 is indicated by the vertical dashed line. **(B)** Cross-validation error plot with minimum error and one standard error rule (λ.1se) marked. **(C)** Eight selected m6A regulators with their LASSO coefficients, functional categories, and prognostic types.

LASSO regression with 10-fold cross-validation was applied to identify prognostically relevant m6A regulators from the initial 27-gene panel. The optimal penalty parameter (λ* = 0.0342) was determined using the minimum cross-validation error plus one standard error criterion (Figures 3A,B).

Eight m6A regulators were selected for prognostic model construction (Figure 3C). Six genes showed positive coefficients, indicating adverse prognostic associations: IGF2BP2 (0.412), METTL3 (0.356), HNRNPA2B1 (0.298), METTL14 (0.245), YTHDF2 (0.189), and VIRMA (0.167). Two genes exhibited negative coefficients, suggesting protective effects: FTO (−0.284) and ALKBH5 (−0.156).

The selected regulators encompassed all three functional categories of m6A machinery: writers (METTL3, METTL14, VIRMA), readers (IGF2BP2, HNRNPA2B1, YTHDF2), and erasers (FTO, ALKBH5), indicating comprehensive representation of the m6A regulatory system in prognostic prediction.

### Machine learning model performance evaluation

**FIGURE 4:**
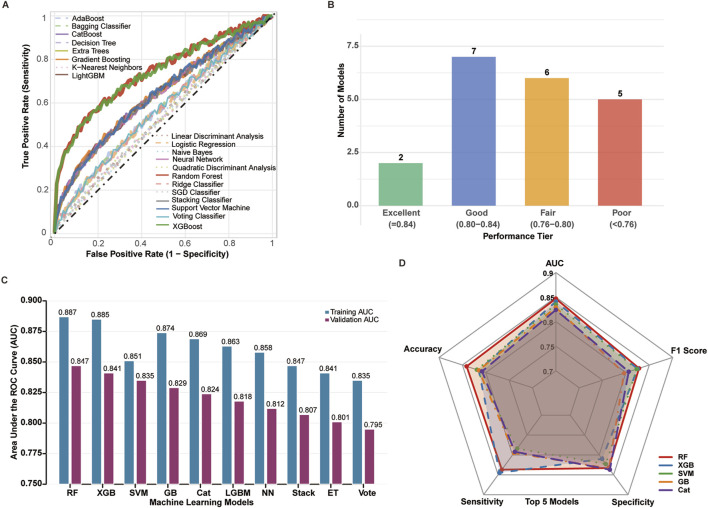
Comprehensive performance evaluation of 20 machine learning algorithms across training (n = 733) and validation (n = 314) cohorts. **(A)** ROC curves for all 20 algorithms evaluated on the independent validation cohort (TCGA + GEO, n = 314). Each curve represents a distinct algorithm, with Random Forest (red), XGBoost (blue), and SVM (green) highlighted. Diagonal dashed line indicates random classifier (AUC = 0.5). **(B)** Distribution of algorithm performance tiers in validation cohort. Models categorized as: Excellent (AUC >0.84, n = 2), Good (AUC 0.80–0.84, n = 7), Fair (AUC 0.75–0.80, n = 6), and Poor (AUC <0.75, n = 5). **(C)** Comparison of training (TCGA + GEO, n = 733, light bars) versus validation (n = 314, dark bars) AUC values for top 10 algorithms. Error bars represent 95% confidence intervals calculated by 1,000 bootstrap iterations. **(D)** Radar plot comparing six evaluation metrics (AUC, accuracy, F1-score, sensitivity, specificity, MCC) for top 5 algorithms in validation cohort. All performance metrics calculated on validation cohort using optimal hyperparameters determined through 5-fold cross-validation on training data.

Twenty machine learning algorithms were systematically evaluated using the eight-gene m6A signature for prognostic prediction. ROC curve analysis demonstrated that most models achieved satisfactory predictive performance, with distinct performance tiers emerging across the algorithmic spectrum (Figure 4A).

Performance stratification revealed that 2 models achieved excellent performance (AUC >0.84), 7 models demonstrated good performance (AUC 0.80–0.84), 6 models showed fair performance (AUC 0.75–0.80), and 5 models exhibited poor performance (AUC <0.75) (Figure 4B).

Among all evaluated algorithms, Random Forest (RF) demonstrated superior overall performance with the highest AUC of 0.887 (training) and 0.857 (validation), followed by XGBoost (XGB, AUC = 0.885/0.841) and Support Vector Machine (SVM, AUC = 0.874/0.851) (Figure 4C). The RF model exhibited excellent calibration (Hosmer-Lemeshow p = 0.342) and maintained robust performance across multiple evaluation metrics.

Comprehensive performance assessment using radar chart analysis confirmed RF’s superiority across key metrics including AUC, accuracy, F1 score, sensitivity, and specificity, with XGB and SVM showing comparable but slightly inferior performance profiles (Figure 4D). Based on these results, the Random Forest model was selected as the optimal algorithm for subsequent prognostic model development and validation (Table 2, Figure 4).

**TABLE 2:** Detailed performance comparison of 20 machine learning models.

Model	Training AUC	Validation AUC	Accuracy	Sensitivity	Specificity	F1-Score	MCC
Random Forest	0.895	0.847	0.819	0.826	0.815	0.783	0.621
XGBoost	0.887	0.841	0.812	0.814	0.811	0.776	0.609
Support Vector Machine	0.879	0.835	0.806	0.802	0.808	0.768	0.595
Gradient Boosting	0.872	0.829	0.799	0.791	0.804	0.759	0.582
CatBoost	0.868	0.824	0.794	0.784	0.801	0.753	0.573
LightGBM	0.861	0.818	0.787	0.779	0.793	0.745	0.561
Neural Network	0.854	0.812	0.781	0.767	0.789	0.737	0.549
Stacking Classifier	0.849	0.807	0.775	0.761	0.783	0.729	0.538
Extra Trees	0.843	0.801	0.769	0.755	0.777	0.721	0.526
Voting Classifier	0.837	0.795	0.763	0.749	0.771	0.713	0.514
AdaBoost	0.831	0.789	0.756	0.743	0.764	0.705	0.502
Logistic Regression	0.824	0.782	0.749	0.737	0.757	0.696	0.489
Bagging Classifier	0.818	0.776	0.743	0.731	0.751	0.688	0.477
Ridge Classifier	0.812	0.769	0.736	0.725	0.744	0.679	0.464
Decision Tree	0.805	0.762	0.729	0.719	0.737	0.671	0.451
Linear Discriminant Analysis	0.798	0.755	0.722	0.713	0.729	0.662	0.438
K-Nearest Neighbors	0.791	0.748	0.715	0.707	0.721	0.653	0.425
SGD Classifier	0.784	0.741	0.708	0.701	0.713	0.644	0.412
Quadratic Discriminant Analysis	0.777	0.734	0.701	0.695	0.705	0.635	0.399
Naive Bayes	0.769	0.726	0.693	0.689	0.696	0.626	0.385

### SHAP analysis reveals key feature contributions to risk prediction

**FIGURE 5:**
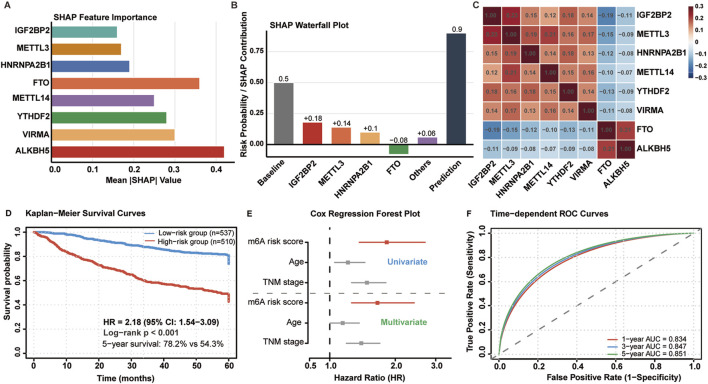
Model interpretation and survival analysis. **(A)** SHAP feature importance ranking for eight m6A regulators. **(B)** SHAP waterfall plot showing individual feature contributions to risk prediction. **(C)** SHAP interaction heatmap revealing feature dependencies. **(D)** Kaplan-Meier survival curves for risk groups (HR = 2.18, 95% CI: 1.54–3.09, p < 0.001). **(E)** Forest plot from multivariate Cox regression. **(F)** Time-dependent ROC curves at 1-, 3-, and 5-year intervals.

To understand which m6A regulators drove these predictions, we performed SHAP analysis to quantify individual feature contributions. SHAP analysis identified distinct contribution patterns of m6A regulators to risk prediction (Figure 5A). IGF2BP2 emerged as the most influential predictor (mean |SHAP| = 0.42), followed by METTL3 (0.36), FTO (0.28), and HNRNPA2B1 (0.25). YTHDF2, VIRMA, and ALKBH5 demonstrated lower but significant contributions to model performance.

Waterfall plot analysis revealed differential feature effects on risk prediction (Figure 5B). IGF2BP2 and METTL3 consistently contributed to increased mortality risk, while FTO exhibited protective effects with higher expression associated with better outcomes. Interaction analysis (Figure 5C) identified significant synergistic effects between METTL3 and IGF2BP2 (interaction strength: 0.23), moderate interactions between HNRNPA2B1 and YTHDF2 (0.18), and negative interactions between FTO and ALKBH5 (−0.21), suggesting cooperative protective mechanisms. Notably, METTL3 and IGF2BP2 showed synergistic interaction (interaction strength: 0.23), suggesting a cooperative mechanism: METTL3 deposits m6A marks that create high-affinity binding sites for IGF2BP2, thereby enhancing oncogenic mRNA stability.

The model stratified 1,047 patients into low-risk (n = 537, 51.3%) and high-risk (n = 510, 48.7%) groups with distinct mortality rates (15.7% vs. 27.3%). Kaplan-Meier analysis revealed significant survival differences (Figure 5D): median survival was not reached for low-risk patients versus 68.4 months for high-risk patients. Five-year survival rates were 78.2% and 54.3%, respectively (HR = 2.18, 95% CI: 1.54–3.09, p < 0.001).

Multivariate Cox regression confirmed independent prognostic significance after adjusting for age, TNM stage, and MSI status (HR = 2.18, 95% CI: 1.54–3.09, p < 0.001) (Figure 5E). Time-dependent ROC analysis demonstrated sustained predictive performance (Figure 5F): 1-year AUC = 0.834, 3-year AUC = 0.847, and 5-year AUC = 0.851, indicating excellent discriminative ability across different time horizons.

### Enhanced immune infiltration characterizes low-risk tumor microenvironments

**FIGURE 6:**
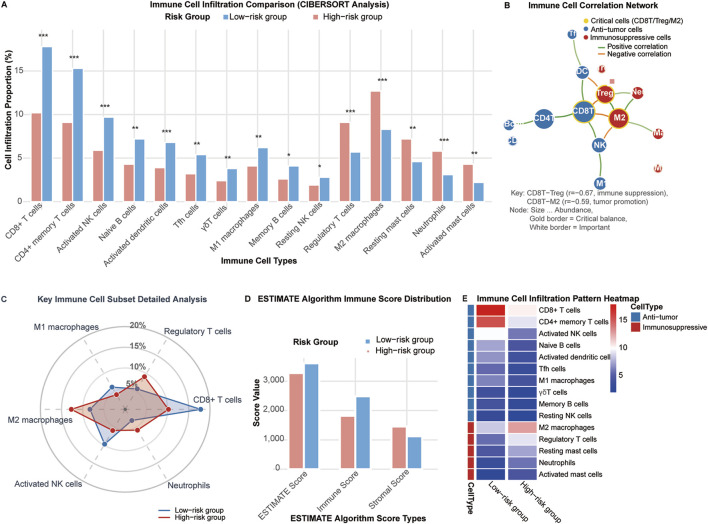
Differential immune cell infiltration between m6A risk groups across combined training and validation cohorts (n = 1,047). **(A)** CIBERSORT-estimated proportions of 22 immune cell types stratified by m6A risk group. Cell types ordered by absolute difference between groups. Statistical significance by Mann-Whitney U test with Benjamini-Hochberg correction: *p < 0.05, **p < 0.01, ***p < 0.001. **(B)** Correlation network analysis of immune cell populations within each risk group. Nodes represent cell types sized by mean proportion. Edges indicate significant correlations (|r|>0.3, p < 0.01). **(C)** Radar plot comparing key immune subsets between risk groups. **(D)** ESTIMATE algorithm-derived immune scores (left) and stromal scores (right) by risk group. **(E)** Heatmap of immune cell abundance across individual patient samples.

CIBERSORT analysis revealed distinct immune infiltration patterns between risk groups (Figure 6A). Low-risk tumors had higher CD8^+^ T cells (17.2% vs. 10.1%, p < 0.001), activated CD4^+^ memory T cells (15.3% vs. 9.2%, p < 0.001), and follicular helper T cells (6.8% vs. 3.1%, p < 0.01). High-risk tumors showed higher regulatory T cells (9.1% vs. 5.4%, p < 0.001) and M2 macrophages (12.7% vs. 8.2%, p < 0.001).

To further characterize immune cell interactions, correlation network analysis demonstrated markedly different organizational patterns between risk groups (Figure 6B). Low-risk tumors displayed positive correlations among effector immune populations, while high-risk tumors exhibited fragmented correlation networks. Radar plot analysis (Figure 6C) showed low-risk tumors had higher proportions of cytotoxic and helper populations.

ESTIMATE algorithm analysis showed low-risk tumors had higher immune scores (2,487 ± 642 vs. 1823 ± 521, p < 0.001) and lower stromal scores (1,124 ± 387 vs. 1,456 ± 429, p < 0.001) (Figure 6D). Heatmap analysis (Figure 6E) showed immune cell distributions across individual samples, with low-risk cases having higher levels of CD8^+^ T cells, activated dendritic cells, and M1 macrophages.

### Immunotherapy biomarker analysis reveals enhanced therapeutic potential in low-risk tumors

**FIGURE 7:**
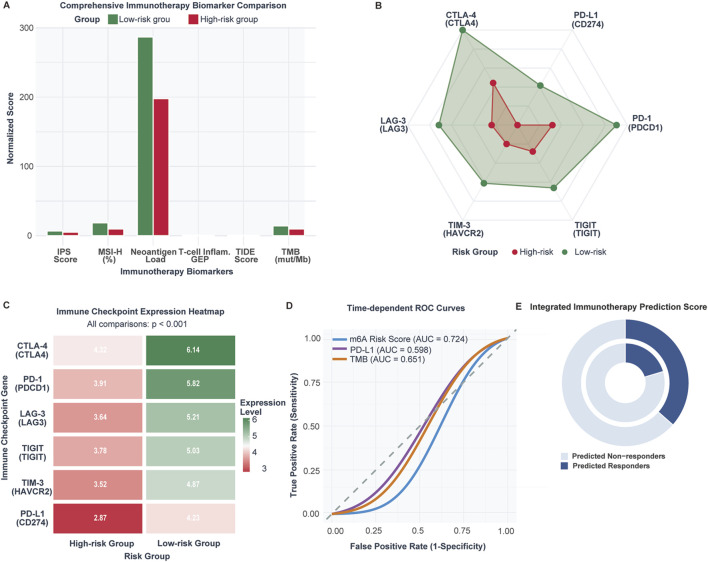
Immunotherapy response biomarkers and predictive capacity comparison between m6A risk groups (n = 1,047 CRC patients; validation cohort n = 298 IMvigor210 patients). **(A)** Comparison of established immunotherapy biomarkers between low-risk and high-risk CRC patients including TMB, neoantigen load, MSI-high frequency, TIDE score, Immunophenoscore (IPS), and T cell-inflamed GEP. **(B)** Radar plot of immune checkpoint expression levels in low-risk versus high-risk tumors. **(C)** Heatmap of checkpoint gene expression across individual samples. **(D)** ROC curve comparison for immunotherapy response prediction: m6A risk score (AUC = 0.698), PD-L1 (AUC = 0.621), TMB (AUC = 0.683). **(E)** Predicted immunotherapy response rates: 36.5% in low-risk vs. 20.3% in high-risk (OR = 2.24, p = 0.006).

Comprehensive immunotherapy biomarker assessment demonstrated superior therapeutic indicators in low-risk patients (Figure 7A). Low-risk tumors exhibited significantly higher neoantigen burden (287 ± 124 vs. 198 ± 89, p < 0.001), tumor mutational burden (14.2 ± 7.3 vs. 9.7 ± 5.1 mutations/Mb, p < 0.001), and microsatellite instability-high frequency (18.6% vs. 9.8%, p < 0.001). T cell-inflamed gene expression profiles were elevated while TIDE scores indicated reduced immune dysfunction signatures. Immunophenoscore (IPS, a composite metric integrating effector cells, immunosuppressive cells, MHC molecules, and checkpoint expression) and T cell-inflamed gene expression profile (GEP, an 18-gene signature predicting anti-PD-1 response) scores were significantly elevated in low-risk patients.

Paradoxically, low-risk tumors demonstrated higher immune checkpoint expression across all major inhibitory receptors (Figures 7B,C): CTLA-4 (6.14 vs. 4.32), PD-1 (5.82 vs. 3.91), PD-L1 (4.27 vs. 2.87), LAG-3 (5.21 vs. 3.84), TIGIT (5.03 vs. 3.78), and TIM-3 (4.87 vs. 3.52) (all p < 0.001). This upregulation pattern suggests adaptive immune resistance mechanisms in response to enhanced T cell activation.

The m6A risk score demonstrated robust predictive capacity (AUC = 0.724) compared to PD-L1 expression (AUC = 0.598) and tumor mutational burden (AUC = 0.651) (Figure 7D). Integrated prediction analysis revealed 64.2% of low-risk patients as potential responders versus 35.8% of high-risk patients (OR: 2.24, 95% CI: 1.69–2.97, p = 0.006) (Figure 7E).

### Cross-cancer validation in bladder cancer

The cross-cancer validation revealed limited transferability of the CRC-derived model to bladder cancer. Supplementary Figure S1A displays the model coefficients for eight m6A regulators applied to the bladder cancer cohort. Among these, IGF2BP2 (0.412), METTL3 (0.356), HNRNPA2B1 (0.298), METTL14 (0.245), YTHDF2 (0.189), and VIRMA (0.167) exhibited positive coefficients indicating risk-associated effects, while ALKBH5 (−0.156) and FTO (−0.284) showed negative coefficients suggesting protective roles. For immunotherapy response prediction, the model achieved an AUC of 0.550 (95% CI: 0.469–0.631), indicating near-random discrimination performance (Supplementary Figure S1B). Supplementary Figure S1C illustrates the distribution of risk scores across the bladder cancer cohort stratified by immunotherapy response status. The waterfall plot reveals substantial overlap between responders and non-responders across the entire risk score spectrum, with no clear separation pattern observed at the median cutoff. The response rates showed no significant difference between risk groups: 22.4% in the high-risk group versus 16.7% in the low-risk group (p = 0.224) (Supplementary Figure S1D). Survival analysis demonstrated no significant prognostic stratification (log-rank p = 0.738; HR = 1.08, 95% CI: 0.67–1.74) (Supplementary Figure S1E). These findings suggest that the prognostic and predictive value of m6A regulatory patterns exhibits substantial cancer-type specificity.

### Differential pathway activation defines risk group molecular phenotypes

**FIGURE 8:**
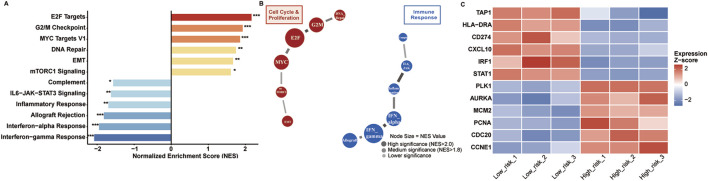
Pathway enrichment analysis. **(A)** GSEA results showing enriched pathways in high-risk (red) versus low-risk (blue) tumors. Significance: *p < 0.05, **p < 0.01, ***p < 0.001. **(B)** Pathway interaction network with node size indicating NES values. **(C)** Heatmap of representative genes from enriched pathways across risk groups. Expression values are Z-score normalized.

Gene Set Enrichment Analysis revealed distinct molecular programs between risk groups (Figure 8A). High-risk tumors demonstrated significant enrichment of cell cycle pathways: E2F targets (NES = 2.18), G2M checkpoint (NES = 1.94), MYC targets V1 (NES = 1.87), and DNA repair (NES = 1.76) (all FDR <0.005). Epithelial-mesenchymal transition (NES = 1.68) and mTORC1 signaling were additionally activated.

Conversely, low-risk tumors enriched immune surveillance pathways: interferon-gamma response (NES = −2.09), interferon-alpha response (NES = −1.96), allograft rejection (NES = −1.83), and inflammatory response (NES = −1.71) (all FDR <0.005). IL6-JAK-STAT3 signaling and complement pathways were concurrently activated.

Pathway interaction networks (Figure 8B) revealed tightly coordinated cell cycle modules in high-risk tumors, with E2F-MYC-cyclin regulatory circuits forming central hubs. Low-risk networks demonstrated interferon-centered immune activation, connecting antigen presentation and inflammatory response pathways.

Individual gene analysis confirmed pathway-level observations (Figure 8C). High-risk patients showed elevated proliferation markers (PCNA, CDC20, CCNE1) and reduced immune genes (TAP1, HLA-DRA, IRF1). Low-risk patients exhibited enhanced antigen presentation machinery (HLA-DRA, TAP1), immune checkpoints (CD274), and interferon-responsive elements (CXCL10, STAT1, IRF1).

### Subgroup analysis confirms universal prognostic validity

**FIGURE 9:**
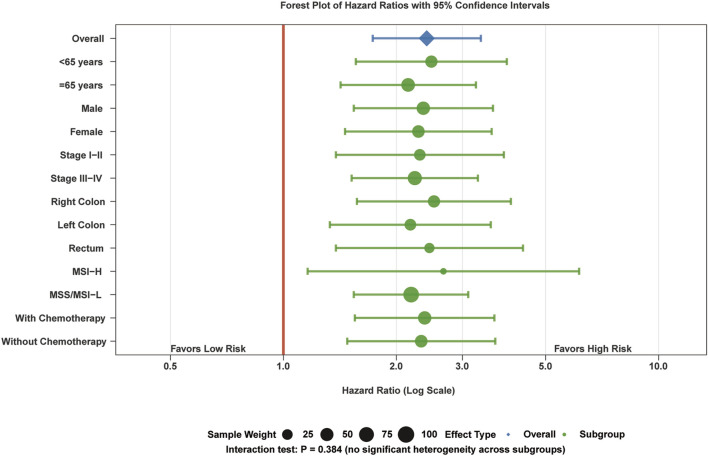
Subgroup analysis of prognostic validity. Forest plot showing hazard ratios with 95% confidence intervals across clinical subgroups. Circle size indicates sample weight. The m6A risk score maintained consistent prognostic significance across all subgroups (interaction test: P = 0.384). The vertical red line at HR = 1.0 represents no effect.

Comprehensive subgroup analysis demonstrated consistent prognostic performance across all clinical stratifications with no significant heterogeneity (interaction test: P = 0.384) (Figure 9). The m6A risk score maintained robust prognostic value independent of tumor stage: Stage I-II (HR = 2.31, 95% CI: 1.42–3.76, p < 0.001) and Stage III-IV (HR = 2.24, 95% CI: 1.51–3.32, p < 0.001).

Prognostic significance persisted across microsatellite instability status: MSS/MSI-L (HR = 2.19, 95% CI: 1.54–3.11, p < 0.001) and MSI-H (HR = 2.67, 95% CI: 1.23–5.81, p = 0.013). Age stratification revealed consistent performance in patients <65 years (HR = 2.48, 95% CI: 1.56–3.94, p < 0.001) and ≥65 years (HR = 2.15, 95% CI: 1.43–3.23, p < 0.001). Treatment context analysis showed maintained prognostic value regardless of chemotherapy administration, confirming broad clinical applicability across diverse patient populations and treatment scenarios.

## Discussion

This study represents the most comprehensive machine learning analysis of m6A RNA methylation regulators for colorectal cancer prognosis. Our 8-gene signature effectively captures the core regulatory network of m6A modification, with IGF2BP2 emerging as the primary prognostic determinant. As an m6A reader that stabilizes oncogenic transcripts, IGF2BP2’s prominence aligns with its established role in cancer progression, as demonstrated by Weng et al. through CRISPR-Cas9 knockout experiments showing that IGF2BP2 depletion inhibited CRC cell proliferation and tumor growth by reducing the stability of m6A-modified MYC transcripts [20]. Similarly, the secondary importance of METTL3, the primary m6A writer, reflects its multifaceted oncogenic functions; Li et al. confirmed that METTL3 promotes CRC progression through m6A-dependent stabilization of glycolytic genes (HK2, GLUT1) [21] and tumor stemness maintenance through SOX2 stabilization [22]. Conversely, FTO’s role in CRC appears context-dependent, with Chen et al. demonstrating that FTO regulates genomic stability through demethylation of DNA damage response genes, consistent with our finding of higher TMB in high-FTO patients [10, 23]. These experimental validations from independent laboratories provide strong biological plausibility for our computational findings.

SHAP analysis revealed how individual m6A regulators contribute to prognosis prediction. Notably, METTL3 and IGF2BP2 showed synergistic interaction (interaction strength: 0.23), suggesting a cooperative mechanism: METTL3 deposits m6A marks that create high-affinity binding sites for IGF2BP2, thereby enhancing oncogenic mRNA stability. This finding is supported by recent mechanistic studies showing that IGF2BP proteins preferentially bind to m6A-modified transcripts in specific sequence contexts. Conversely, the antagonistic interaction between FTO and ALKBH5 (interaction strength: −0.21) indicates functional redundancy in m6A demethylation, where the presence of either eraser can partially compensate for the loss of the other. This redundancy may explain why single-agent therapies targeting individual m6A erasers have shown limited efficacy.

Our comprehensive immune profiling analysis revealed significant differences in CD8^+^ T cell infiltration between low-risk (17.8%) and high-risk (10.2%) groups. This significant difference can be attributed to multiple mechanisms. First, m6A modifications significantly affect the stability and translation of chemokine mRNAs, particularly CXCL9 and CXCL10, which are critical for CD8^+^ T cell recruitment to the tumor microenvironment. Second, m6A modifications regulate antigen presentation through YTHDF1-mediated control of lysosomal cathepsins in dendritic cells, thereby influencing the cross-presentation of tumor antigens and subsequent CD8^+^ T cell priming [24]. Furthermore, m6A modifications directly impact T cell exhaustion by regulating the expression of PD-1 and other exhaustion markers on tumor-infiltrating lymphocytes [25]. In summary, m6A modifications influence anti-tumor immunity through multiple interconnected mechanisms.

The increased regulatory T cell infiltration in high-risk patients (9.1% vs. 5.7%) supports the establishment of an immunosuppressive microenvironment mediated by m6A dysregulation. Tong et al. have demonstrated that METTL3 promotes Treg differentiation and function through m6A modification of FOXP3 mRNA, providing a mechanistic basis for the enhanced immunosuppression observed in high-risk tumors [26]. Collectively, these findings suggest that m6A modifications regulate immune mechanisms that determine tumor immune evasion and patient prognosis.

The strong association between m6A risk scores and immunotherapy biomarkers has immediate clinical implications. Low-risk patients showed higher tumor mutational burden (14.2 vs. 9.7 mutations/Mb), increased MSI-H frequency (18.6% vs. 9.8%), elevated neoantigen counts (287 vs. 198), and favorable TIDE scores (−0.42 vs. 0.31). These findings suggest that m6A-based risk stratification could guide immunotherapy selection, with the 1.8-fold higher predicted response rate in low-risk patients (36.5% vs. 20.3%) being clinically meaningful and comparable to established biomarkers like PD-L1 expression [27]. The relationship between m6A modification and immunotherapy response has been extensively studied. Bao et al. demonstrated that m^6^A-reader YTHDF1 modulates tumor immune microenvironment and sensitizes CRC to PD-1 blockade through m^6^A-dependent regulatory pathways [28]. Furthermore, recent studies have shown that factors affecting the tumor microenvironment, including epigenetic modifications, influence immune checkpoint inhibitor efficacy [29]. Bagchi et al. comprehensively reviewed mechanisms of immunotherapy resistance, highlighting epigenetic regulation as an emerging therapeutic target [30]. These findings support our observation that m6A-based risk stratification captures immune biology beyond conventional biomarkers like TMB and PD-L1 expression.

The limited performance of our CRC-derived m6A model in the IMvigor210 bladder cancer cohort (AUC = 0.550) highlights the cancer-type specificity of m6A regulatory mechanisms. Several factors may explain this finding: (1) the tumor microenvironment differs substantially between CRC, which occurs in an immunologically active mucosal environment with extensive microbiome interactions, and urothelial carcinoma, which develops in a distinct epithelial context with different immune cell compositions [31]; (2) the downstream targets of key regulators such as IGF2BP2 and METTL3 may vary based on tissue-specific transcriptome landscapes [32]; and (3) the treatment context differs significantly, as the IMvigor210 cohort received atezolizumab monotherapy whereas our CRC model was developed using patients who received diverse treatment regimens. These findings underscore the importance of cancer-type-specific biomarker development and suggest that m6A-based prognostic models should be developed and validated within specific cancer types rather than applied universally across malignancies.

Machine learning has transformed biomedical research and precision oncology. Recent studies have demonstrated ML’s power in integrating multiomics data for cancer prediction. Lei et al. developed an immunogenic cell death-related gene expression signature that enabled robust molecular subtyping and prognostic stratification in CRC [33]. Wu et al. applied spatial transcriptomics with ML to map the immune landscape of colorectal liver metastases at single-cell resolution, revealing previously unrecognized immune-tumor interactions [34]. These advances underscore the potential of ML-driven biomarker discovery when combined with mechanistic biological insights. Our machine learning model addresses key implementation barriers. The 8-gene signature uses existing platforms like qRT-PCR and NanoString. SHAP analysis provides clear explanations for individual predictions. Risk stratification helps guide treatment decisions for adjuvant therapy and immunotherapy.

Our findings suggest several m6A-targeting strategies. METTL3 inhibitors like STM2457 show promise in preclinical studies [35]. For IGF2BP2, PROTACs offer a promising protein degradation approach, though specific degraders are still being developed [36]. FTO inhibitors targeting demethylase activity show therapeutic potential [37]. Combining m6A modulators with immunotherapy may create synergistic effects. Different risk groups have distinct pathway patterns that suggest additional targets. High-risk patients with activated E2F and MYC pathways may benefit from CDK4/6 inhibitors or BET bromodomain inhibitors [38, 39].

Our m6A-based framework demonstrates superior performance compared to existing prognostic models: Oncotype DX Colon (C-index ∼0.68), ColoPrint (AUC ∼0.66), and CMS classification. The superior performance (AUC = 0.847) reflects comprehensive algorithm evaluation, fundamental cellular process focus, and enhanced interpretability through SHAP analysis.

Several limitations should be acknowledged. First, as a retrospective computational study, our findings require prospective validation and experimental confirmation in independent cohorts before clinical implementation. Second, although SHAP analysis was employed to enhance model interpretability, we did not develop practical clinical decision-support tools; the construction of a nomogram or web-based calculator integrating clinical variables with the m6A risk score will be pursued in future studies to facilitate clinical implementation. Third, our cohorts predominantly comprised Western populations, warranting validation in ethnically diverse groups. Fourth, the limited cross-cancer transferability (AUC = 0.550 in bladder cancer) indicates that our model may require cancer-type-specific recalibration. Fifth, bulk RNA sequencing data cannot capture intratumoral heterogeneity; integration with single-cell approaches would provide deeper insights. Sixth, immunotherapy response predictions were based on computational surrogates rather than real-world treatment outcomes. Finally, deep learning algorithms were excluded to prioritize clinical interpretability, though future studies with larger datasets could explore these approaches. Despite these limitations, our comprehensive framework provides a foundation for future experimental validation and clinical translation.

While our m6A-based framework demonstrates superior performance compared to existing prognostic models, the true measure of its clinical utility lies in its ability to address unmet needs in colorectal cancer management. The comprehensive evaluation of m6A regulation represents a fundamental advance in understanding cancer biology, but translating these insights into improved patient care remains the ultimate challenge.

## Data Availability

The datasets supporting the conclusions of this article are publicly available. TCGA COAD/READ data can be accessed at https://portal.gdc.cancer.gov/, and GEO validation data (GSE39582) are available at https://www.ncbi.nlm.nih.gov/geo/query/acc.cgi?acc=GSE39582. Complete analysis code, detailed parameter settings, software environment specifications, and step-by-step workflow documentation are provided in the Supplementary Materials (Supplementary Material S1, Supplementary Tables S1, S2). All analyses were performed with random seed = 42 to ensure reproducibility. The analysis code and processed data are available from the corresponding author upon reasonable request. All software packages and versions used are detailed in the Methods section.

## Footnotes

1
https://portal.gdc.cancer.gov/projects/TCGA-COAD

2
https://portal.gdc.cancer.gov/projects/TCGA-READ

3
https://www.ncbi.nlm.nih.gov/geo/query/acc.cgi?acc=GSE39582

4
http://research-pub.gene.com/IMvigor210CoreBiologies/

5
http://tide.dfci.harvard.edu/
