# Supplementary figures and images for: Machine learning-based comprehensive analysis of m6A RNA methylation regulators in colorectal cancer: implications for prognosis, immune microenvironment, and immunotherapy response
Source: Exp Biol Med (Maywood). 2026 Jan 14;250:10776. doi: 10.3389/ebm.2025.10776 (PMC12847061; doi:10.3389/ebm.2025.10776)

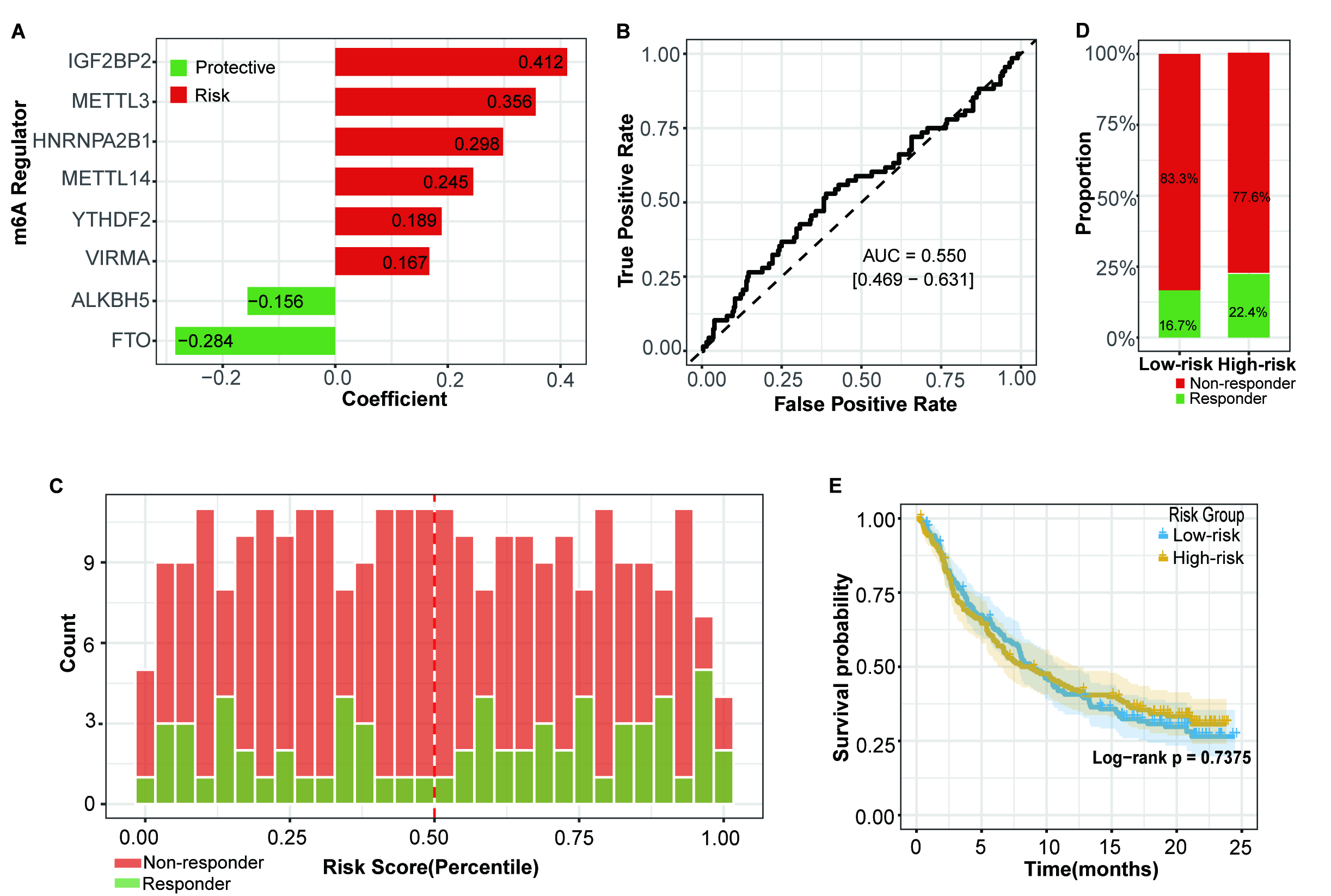

Supplement: Supplementary file 3 [file Image1.tif]
